# 9-Hy­droxy-4,8-dimethyl-12-(pyrrolidin-1-ylmeth­yl)-3,14-dioxatricyclo­[9.3.0.0^2,4^]tetra­dec-7-en-13-one

**DOI:** 10.1107/S1600536811030467

**Published:** 2011-08-02

**Authors:** Mohamed Moumou, Ahmed Benharref, Daniel Avignant, Abdelghani Oudahmane, Mohamed Akssira, Moha Berraho

**Affiliations:** a Laboratoire de Chimie Biomoleculaire, Substances Naturelles et Réactivité, URAC16, Faculté des Sciences Semlalia, BP 2390 Bd My Abdellah, 40000 Marrakech, Morocco; b Laboratoire des Matériaux Inorganiques, Université Blaise Pascal, UMR CNRS 6002, 24 Avenue des Landais, 63177 Aubière, France; c Laboratoire de Chimie Bioorganique et Analytique, URAC 22, BP 146, FSTM, Université Hassan II, Mohammedia–Casablanca 20810 Mohammedia, Morocco

## Abstract

The title compound, C_19_H_29_O_4_, was synthesized from 9α-hy­droxy­parthenolide (9α-hy­droxy-4,8-dimethyl-12-methylen-3,14-dioxatricyclo­[9.3.0.0^2,4^]tetra­dec-7-en-13-one), which was isolated from the chloro­form extract of the aerial parts of *Anvillea radiata*. The mol­ecule is built up from two fused five- and ten-membered rings with the pyrrolidin-1-ylmethyl group as a substituent. The five-membered lactone ring has an envelope conformation, whereas the ten-membered and pyrrolidine rings display approximate chair–chair and twisted conformations, respectively. The dihedral angle between the ten-membered ring and the lactone ring is 18.01 (19)°. An intra­molecular O—H⋯N hydrogen bond occurs. The crystal structure is stabilized by weak inter­molecular C—H⋯O hydrogen-bonding inter­actions.

## Related literature

For the isolation and biological activity of 9α-hy­droxy­parthenolide, see: Abdel Sattar *et al.* (1996 ▶); El Hassany *et al.* (2004 ▶). For the reactivity of this sesquiterpene, see: Castaneda-Acosta *et al.* (1993 ▶); Neukirch *et al.* (2003 ▶); Der-Ren *et al.* (2006 ▶); Neelakantan *et al.* (2009 ▶). For conformational analysis, see: Cremer & Pople (1975 ▶)
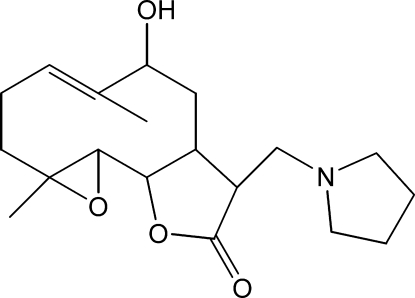


## Experimental

### 

#### Crystal data

C_19_H_29_NO_4_
*M* *_r_* = 335.43
Orthorhombic, 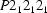

*a* = 8.1389 (6) Å
*b* = 10.1788 (7) Å
*c* = 21.7669 (15) Å
*V* = 1803.3 (2) Å^3^
*Z* = 4
Mo *K*α radiation
μ = 0.09 mm^−1^
*T* = 298 K
0.30 × 0.27 × 0.18 mm

#### Data collection

Bruker APEXII CCD area-detector diffractometer
Absorption correction: multi-scan (*SADABS*; Bruker, 2008 ▶) *T*_min_ = 0.634, *T*_max_ = 0.746
7656 measured reflections
2110 independent reflections
1220 reflections with *I* > 2σ(*I*)
*R*_int_ = 0.053

#### Refinement

*R*[*F* ^2^ > 2σ(*F* ^2^)] = 0.045
*wR*(*F* ^2^) = 0.121
*S* = 0.99
2110 reflections
222 parameters
H-atom parameters constrained
Δρ_max_ = 0.15 e Å^−3^
Δρ_min_ = −0.15 e Å^−3^

### 

Data collection: *APEX2* (Bruker, 2005 ▶); cell refinement: *APEX2* and *SAINT* (Bruker, 2005 ▶); data reduction: *SAINT*; program(s) used to solve structure: *SHELXS97* (Sheldrick, 2008 ▶); program(s) used to refine structure: *SHELXL97* (Sheldrick, 2008 ▶); molecular graphics: *ORTEP-3 for Windows* (Farrugia, 1997 ▶) and *PLATON* (Spek, 2009 ▶); software used to prepare material for publication: *WinGX* (Farrugia, 1999 ▶).

## Supplementary Material

Crystal structure: contains datablock(s) I, global. DOI: 10.1107/S1600536811030467/zl2390sup1.cif

Structure factors: contains datablock(s) I. DOI: 10.1107/S1600536811030467/zl2390Isup2.hkl

Supplementary material file. DOI: 10.1107/S1600536811030467/zl2390Isup3.cml

Additional supplementary materials:  crystallographic information; 3D view; checkCIF report

## Figures and Tables

**Table 1:** Hydrogen-bond geometry (Å, °)

*D*—H⋯*A*	*D*—H	H⋯*A*	*D*⋯*A*	*D*—H⋯*A*
O4—H4⋯N	0.82	2.17	2.964 (4)	164
C1—H1⋯O4^i^	0.98	2.57	3.533 (4)	167
C11—H11⋯O1^ii^	0.98	2.50	3.403 (4)	154

Symmetry codes: (i) 
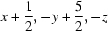
; (ii) 
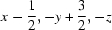
.

**Fig. 1.**
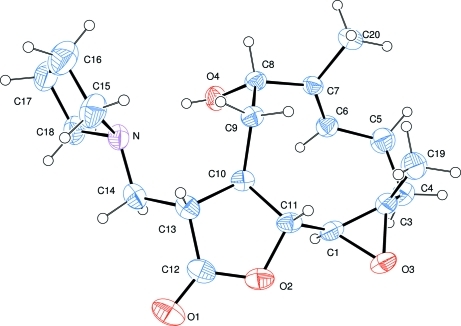
: Molecular structure of the title compound with the atom-labelling scheme. Displacement ellipsoids are drawn at the 30% probability level. H atoms are represented as small spheres of arbitrary radii.

**Fig. 2.**
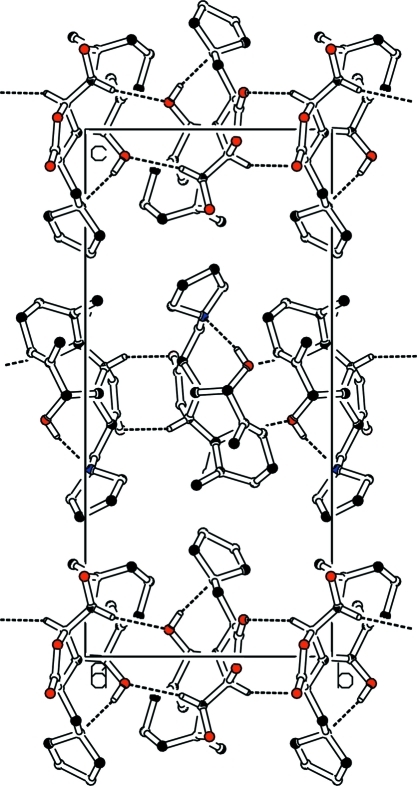
: Packing view showing the C–H···O and O–H···N hydrogen bonds as dashed lines. H atoms not involved in hydrogen bonding have been omitted for clarity.

## supplementary crystallographic information

### Comment

The natural sesquiterpene lactone 9α-hydroxypartenolide is the main constituent of the chloroform extract of the aerial parts of *Anvillea radiata* (El Hassany *et al.*, 2004) and of *Anvillea garcini* (Abdel Sattar *et al.*, 1996). The reactivity of this sesquiterpene lactone and its derivatives has been the subject of several studies (Castaneda-Acosta *et al.*, 1993; Neukirch *et al.*, 2003; Der-Ren *et al.*, 2006; Neelakantan *et al.*, 2009), with the aim to prepare products with a high added value that can be used in the pharmacological industry. In the same context, we have treated 9α-hydroxyparthenolide with one equivalent of pyrrolidine and obtained 9-hydroxy-4,8-dimethyl-12-pyrrolidin-1-ylmethyl-3, 14-dioxatricyclo[9.3.0.0^2,4^] tetradec -7-en-13-one with a good yield of 84%. The structure of this new derivative of 9α-hydroxypartenolide was determined by its single-crystal X-ray structure. The molecule contains two fused rings which exhibit different conformations with a pyrrolidine ring as a substituent to the lactone ring. The molecular structure of the title compound, Fig.1, shows the lactone ring to adopt an envelope conformation, as indicated by Cremer & Pople (1975) puckering parameters QT = 0.291 (4) Å and φ2 = 78.1 (7)°. The ten-membered ring displays an approximate chair-chair conformation, while the pyrrolidine ring has a twisted conformation with QT = 0.377 (4) Å, φ2 = 15.0 (8)°. In the crystal structure, molecules are connected through C—H···O hydrogen bonds, forming chains running along the *b* axis. (Fig.2). In addition an intramolecular O—H···N hydrogen bond is also observed.

### Experimental

A mixture of 9α-hydoxypartenolide (300 mg, 1.13 mmol) and one equivalent of pyrrolidine in EtOH (20 ml) was stirred for one night at room temperature. The next day the reaction was stopped by adding 10 ml of water and extracted three times with ethyl acetate (3 × 20 ml). The combined organic layers were dried over anhydrous sodium sulfate, filtered and concentrated under vacuum to give 315 mg (0.94 mmol, 84%) of 9-hydroxy-4,8-dimethyl-12-pyrrolidin- 1-ylmethyl-3,14-dioxatricyclo[9.3.0.0^2,4^]tetradec-7-en-13-one. The title compound was recrystallized in ethyl acetate.

### Refinement

Reflections (1 0 2), (1 0 1), (1 1 0), (0 1 3), (0 1 1), (0 1 2) and (1 1 2) were obstructed by the beam stop and were omitted from the refinement. All H atoms were fixed geometrically and treated as riding with C—H = 0.96 Å (methyl), 0.97 Å (methylene), 0.98 Å (methine) and O–H = 0.82 Å with *U*_iso_(H) = 1.2*U*_eq_ (methylene, methine) or *U*_iso_(H) = 1.5*U*_eq_ (methyl, OH). In the absence of significant anomalous scattering, the absolute configuration could not be reliably determined and thus 1554 Friedel pairs were merged and any references to the Flack parameter were removed. The choice of enantiomer is assigned arbitrarily.

### Crystal data

**Table:**

C_19_H_29_NO_4_	*F*(000) = 728
*M**_r_* = 335.43	*D*_x_ = 1.236 Mg m^−^^3^
Orthorhombic, *P*2_1_2_1_2_1_	Mo *K*α radiation, λ = 0.71073 Å
Hall symbol: P 2ac 2ab	Cell parameters from 7656 reflections
*a* = 8.1389 (6) Å	θ = 3.7–26.4°
*b* = 10.1788 (7) Å	µ = 0.09 mm^−^^1^
*c* = 21.7669 (15) Å	*T* = 298 K
*V* = 1803.3 (2) Å^3^	PRISM, colourless
*Z* = 4	0.30 × 0.27 × 0.18 mm

### Data collection

**Table:**

Bruker APEXII CCD area-detector diffractometer	2110 independent reflections
Radiation source: fine-focus sealed tube	1220 reflections with *I* > 2σ(*I*)
graphite	*R*_int_ = 0.053
Detector resolution: 8.3333 pixels mm^-1^	θ_max_ = 26.4°, θ_min_ = 4.0°
φ and ω scans	*h* = −10→9
Absorption correction: multi-scan (*SADABS*; Bruker, 2008)	*k* = −10→12
*T*_min_ = 0.634, *T*_max_ = 0.746	*l* = −16→27
7656 measured reflections	

### Refinement

**Table:**

Refinement on *F*^2^	Secondary atom site location: difference Fourier map
Least-squares matrix: full	Hydrogen site location: inferred from neighbouring sites
*R*[*F*^2^ > 2σ(*F*^2^)] = 0.045	H-atom parameters constrained
*wR*(*F*^2^) = 0.121	*w* = 1/[σ^2^(*F*_o_^2^) + (0.0601*P*)^2^] where *P* = (*F*_o_^2^ + 2*F*_c_^2^)/3
*S* = 0.99	(Δ/σ)_max_ < 0.001
2110 reflections	Δρ_max_ = 0.15 e Å^−^^3^
222 parameters	Δρ_min_ = −0.15 e Å^−^^3^
0 restraints	Extinction correction: *SHELXL97* (Sheldrick, 2008), Fc^*^=kFc[1+0.001xFc^2^λ^3^/sin(2θ)]^-1/4^
Primary atom site location: structure-invariant direct methods	Extinction coefficient: 0.017 (3)

### Special details

**Table:**

Geometry. All s.u.'s (except the s.u. in the dihedral angle between two l.s. planes) are estimated using the full covariance matrix. The cell s.u.'s are taken into account individually in the estimation of s.u.'s in distances, angles and torsion angles; correlations between s.u.'s in cell parameters are only used when they are defined by crystal symmetry. An approximate (isotropic) treatment of cell s.u.'s is used for estimating s.u.'s involving l.s. planes.

### Fractional atomic coordinates and isotropic or equivalent isotropic displacement parameters (Å^2^)

**Table:**

	*x*	*y*	*z*	*U*_iso_*/*U*_eq_	
C1	0.2577 (4)	1.0264 (3)	0.09346 (16)	0.0496 (9)	
H1	0.2982	1.1080	0.0749	0.059*	
C3	0.1673 (5)	1.0469 (4)	0.15028 (16)	0.0548 (10)	
C4	0.1500 (5)	1.1871 (4)	0.16990 (18)	0.0673 (11)	
H4A	0.2481	1.2346	0.1578	0.081*	
H4B	0.1430	1.1901	0.2144	0.081*	
C5	−0.0026 (5)	1.2584 (4)	0.14249 (19)	0.0694 (12)	
H5A	−0.0984	1.2386	0.1674	0.083*	
H5B	0.0151	1.3526	0.1438	0.083*	
C6	−0.0347 (5)	1.2170 (3)	0.07708 (16)	0.0508 (10)	
H6	0.0391	1.2462	0.0475	0.061*	
C7	−0.1572 (4)	1.1435 (3)	0.05791 (15)	0.0427 (8)	
C8	−0.1582 (4)	1.0771 (3)	−0.00454 (15)	0.0462 (9)	
H8	−0.2722	1.0556	−0.0150	0.055*	
C9	−0.0599 (4)	0.9476 (3)	−0.00085 (16)	0.0441 (8)	
H9A	−0.0842	0.9051	0.0380	0.053*	
H9B	−0.0968	0.8897	−0.0334	0.053*	
C10	0.1272 (4)	0.9652 (3)	−0.00631 (15)	0.0428 (8)	
H10	0.1484	1.0592	−0.0118	0.051*	
C11	0.2305 (4)	0.9192 (3)	0.04834 (16)	0.0469 (9)	
H11	0.1811	0.8422	0.0680	0.056*	
C12	0.3823 (5)	0.8774 (3)	−0.0393 (2)	0.0580 (10)	
C13	0.2083 (4)	0.8935 (3)	−0.05999 (17)	0.0519 (10)	
H13	0.1584	0.8063	−0.0639	0.062*	
C14	0.1989 (5)	0.9619 (4)	−0.12159 (17)	0.0604 (11)	
H14A	0.2482	1.0483	−0.1177	0.072*	
H14B	0.2635	0.9126	−0.1511	0.072*	
C15	−0.0436 (6)	0.8528 (4)	−0.1647 (2)	0.0802 (14)	
H15A	−0.0827	0.8038	−0.1293	0.096*	
H15B	0.0333	0.7987	−0.1874	0.096*	
C16	−0.1830 (7)	0.8946 (5)	−0.2044 (3)	0.1017 (17)	
H16A	−0.2811	0.9096	−0.1801	0.122*	
H16B	−0.2067	0.8285	−0.2353	0.122*	
C17	−0.1246 (6)	1.0221 (5)	−0.23440 (19)	0.0808 (14)	
H17A	−0.1037	1.0089	−0.2778	0.097*	
H17B	−0.2065	1.0907	−0.2298	0.097*	
C18	0.0303 (6)	1.0581 (4)	−0.20152 (16)	0.0690 (12)	
H18A	0.1253	1.0398	−0.2270	0.083*	
H18B	0.0303	1.1507	−0.1910	0.083*	
C19	0.0421 (5)	0.9509 (4)	0.17490 (18)	0.0735 (13)	
H19A	0.0628	0.8653	0.1581	0.110*	
H19B	−0.0662	0.9791	0.1634	0.110*	
H19C	0.0499	0.9474	0.2189	0.110*	
C20	−0.3045 (5)	1.1034 (4)	0.09593 (18)	0.0682 (12)	
H20A	−0.2922	1.1361	0.1370	0.102*	
H20B	−0.3124	1.0093	0.0969	0.102*	
H20C	−0.4025	1.1393	0.0780	0.102*	
N	0.0336 (4)	0.9768 (3)	−0.14550 (13)	0.0539 (8)	
O1	0.5035 (4)	0.8578 (3)	−0.06984 (15)	0.0824 (9)	
O2	0.3922 (3)	0.8876 (2)	0.02235 (13)	0.0600 (7)	
O3	0.3332 (3)	0.9963 (3)	0.15201 (12)	0.0662 (8)	
O4	−0.0948 (3)	1.1599 (2)	−0.05115 (11)	0.0544 (7)	
H4	−0.0741	1.1161	−0.0818	0.060 (13)*	

### Atomic displacement parameters (Å^2^)

**Table:**

	*U*^11^	*U*^22^	*U*^33^	*U*^12^	*U*^13^	*U*^23^
C1	0.040 (2)	0.055 (2)	0.054 (2)	−0.0002 (18)	−0.0123 (19)	0.0075 (18)
C3	0.046 (2)	0.071 (3)	0.047 (2)	0.004 (2)	−0.0050 (19)	0.011 (2)
C4	0.059 (3)	0.088 (3)	0.055 (2)	0.000 (2)	−0.009 (2)	−0.010 (2)
C5	0.072 (3)	0.070 (3)	0.066 (3)	0.007 (2)	−0.003 (3)	−0.012 (2)
C6	0.050 (2)	0.049 (2)	0.053 (2)	0.0035 (18)	−0.001 (2)	−0.0002 (17)
C7	0.0356 (19)	0.0471 (19)	0.0454 (19)	0.0053 (16)	0.0035 (18)	0.0030 (17)
C8	0.038 (2)	0.054 (2)	0.046 (2)	−0.0014 (15)	−0.0027 (18)	0.0111 (18)
C9	0.043 (2)	0.0413 (18)	0.0484 (19)	−0.0034 (15)	−0.0035 (17)	0.0050 (17)
C10	0.041 (2)	0.0349 (17)	0.052 (2)	0.0005 (15)	−0.0019 (18)	0.0044 (16)
C11	0.038 (2)	0.0424 (19)	0.060 (2)	0.0016 (15)	−0.0031 (19)	0.0104 (17)
C12	0.055 (3)	0.041 (2)	0.078 (3)	0.0097 (19)	0.009 (3)	−0.0028 (19)
C13	0.051 (2)	0.0458 (19)	0.059 (2)	−0.0019 (17)	0.008 (2)	−0.0038 (18)
C14	0.062 (3)	0.064 (2)	0.056 (2)	0.004 (2)	0.012 (2)	−0.007 (2)
C15	0.106 (4)	0.065 (3)	0.069 (3)	−0.026 (3)	−0.011 (3)	−0.011 (2)
C16	0.111 (4)	0.109 (4)	0.085 (3)	−0.026 (4)	−0.022 (3)	−0.012 (3)
C17	0.091 (4)	0.093 (3)	0.059 (2)	0.003 (3)	−0.012 (3)	−0.012 (2)
C18	0.083 (3)	0.079 (3)	0.045 (2)	−0.009 (3)	0.006 (2)	0.004 (2)
C19	0.067 (3)	0.089 (3)	0.065 (2)	−0.011 (3)	0.002 (2)	0.026 (2)
C20	0.046 (2)	0.095 (3)	0.064 (2)	−0.002 (2)	0.008 (2)	0.001 (2)
N	0.062 (2)	0.0553 (18)	0.0444 (16)	−0.0090 (16)	0.0040 (16)	−0.0051 (15)
O1	0.068 (2)	0.0735 (19)	0.106 (2)	0.0188 (16)	0.0221 (19)	−0.0043 (18)
O2	0.0461 (16)	0.0548 (15)	0.0791 (19)	0.0125 (13)	−0.0057 (14)	0.0001 (13)
O3	0.0478 (16)	0.0875 (19)	0.0634 (16)	0.0074 (14)	−0.0176 (14)	0.0097 (14)
O4	0.0635 (17)	0.0531 (14)	0.0466 (14)	0.0046 (14)	0.0035 (14)	0.0102 (13)

### Geometric parameters (Å, °)

**Table:**

C1—O3	1.448 (4)	C12—O1	1.206 (4)
C1—C3	1.454 (5)	C12—O2	1.349 (5)
C1—C11	1.485 (5)	C12—C13	1.495 (5)
C1—H1	0.9800	C13—C14	1.513 (5)
C3—O3	1.446 (4)	C13—H13	0.9800
C3—C4	1.496 (5)	C14—N	1.450 (4)
C3—C19	1.510 (5)	C14—H14A	0.9700
C4—C5	1.558 (5)	C14—H14B	0.9700
C4—H4A	0.9700	C15—N	1.470 (5)
C4—H4B	0.9700	C15—C16	1.489 (6)
C5—C6	1.508 (5)	C15—H15A	0.9700
C5—H5A	0.9700	C15—H15B	0.9700
C5—H5B	0.9700	C16—C17	1.529 (6)
C6—C7	1.315 (4)	C16—H16A	0.9700
C6—H6	0.9300	C16—H16B	0.9700
C7—C20	1.513 (5)	C17—C18	1.495 (6)
C7—C8	1.518 (5)	C17—H17A	0.9700
C8—O4	1.417 (4)	C17—H17B	0.9700
C8—C9	1.544 (5)	C18—N	1.474 (5)
C8—H8	0.9800	C18—H18A	0.9700
C9—C10	1.538 (4)	C18—H18B	0.9700
C9—H9A	0.9700	C19—H19A	0.9600
C9—H9B	0.9700	C19—H19B	0.9600
C10—C13	1.528 (5)	C19—H19C	0.9600
C10—C11	1.530 (4)	C20—H20A	0.9600
C10—H10	0.9800	C20—H20B	0.9600
C11—O2	1.468 (4)	C20—H20C	0.9600
C11—H11	0.9800	O4—H4	0.8200
			
O3—C1—C3	59.8 (2)	O2—C12—C13	110.3 (3)
O3—C1—C11	119.3 (3)	C12—C13—C14	111.4 (3)
C3—C1—C11	126.3 (3)	C12—C13—C10	103.4 (3)
O3—C1—H1	113.6	C14—C13—C10	115.8 (3)
C3—C1—H1	113.6	C12—C13—H13	108.6
C11—C1—H1	113.6	C14—C13—H13	108.6
O3—C3—C1	59.9 (2)	C10—C13—H13	108.6
O3—C3—C4	114.9 (3)	N—C14—C13	114.4 (3)
C1—C3—C4	115.3 (3)	N—C14—H14A	108.7
O3—C3—C19	113.0 (3)	C13—C14—H14A	108.7
C1—C3—C19	123.4 (4)	N—C14—H14B	108.7
C4—C3—C19	116.9 (4)	C13—C14—H14B	108.7
C3—C4—C5	114.2 (3)	H14A—C14—H14B	107.6
C3—C4—H4A	108.7	N—C15—C16	104.2 (4)
C5—C4—H4A	108.7	N—C15—H15A	110.9
C3—C4—H4B	108.7	C16—C15—H15A	110.9
C5—C4—H4B	108.7	N—C15—H15B	110.9
H4A—C4—H4B	107.6	C16—C15—H15B	110.9
C6—C5—C4	111.7 (3)	H15A—C15—H15B	108.9
C6—C5—H5A	109.3	C15—C16—C17	104.7 (4)
C4—C5—H5A	109.3	C15—C16—H16A	110.8
C6—C5—H5B	109.3	C17—C16—H16A	110.8
C4—C5—H5B	109.3	C15—C16—H16B	110.8
H5A—C5—H5B	107.9	C17—C16—H16B	110.8
C7—C6—C5	126.2 (4)	H16A—C16—H16B	108.9
C7—C6—H6	116.9	C18—C17—C16	105.4 (4)
C5—C6—H6	116.9	C18—C17—H17A	110.7
C6—C7—C20	125.5 (3)	C16—C17—H17A	110.7
C6—C7—C8	122.8 (3)	C18—C17—H17B	110.7
C20—C7—C8	111.4 (3)	C16—C17—H17B	110.7
O4—C8—C7	112.0 (3)	H17A—C17—H17B	108.8
O4—C8—C9	110.9 (3)	N—C18—C17	105.9 (3)
C7—C8—C9	109.3 (3)	N—C18—H18A	110.6
O4—C8—H8	108.2	C17—C18—H18A	110.6
C7—C8—H8	108.2	N—C18—H18B	110.6
C9—C8—H8	108.2	C17—C18—H18B	110.6
C10—C9—C8	114.2 (3)	H18A—C18—H18B	108.7
C10—C9—H9A	108.7	C3—C19—H19A	109.5
C8—C9—H9A	108.7	C3—C19—H19B	109.5
C10—C9—H9B	108.7	H19A—C19—H19B	109.5
C8—C9—H9B	108.7	C3—C19—H19C	109.5
H9A—C9—H9B	107.6	H19A—C19—H19C	109.5
C13—C10—C11	102.2 (3)	H19B—C19—H19C	109.5
C13—C10—C9	115.6 (3)	C7—C20—H20A	109.5
C11—C10—C9	116.6 (3)	C7—C20—H20B	109.5
C13—C10—H10	107.3	H20A—C20—H20B	109.5
C11—C10—H10	107.3	C7—C20—H20C	109.5
C9—C10—H10	107.3	H20A—C20—H20C	109.5
O2—C11—C1	106.4 (3)	H20B—C20—H20C	109.5
O2—C11—C10	105.1 (3)	C14—N—C15	114.1 (3)
C1—C11—C10	111.8 (3)	C14—N—C18	111.9 (3)
O2—C11—H11	111.1	C15—N—C18	103.8 (3)
C1—C11—H11	111.1	C12—O2—C11	110.3 (3)
C10—C11—H11	111.1	C3—O3—C1	60.3 (2)
O1—C12—O2	120.8 (4)	C8—O4—H4	109.5
O1—C12—C13	128.9 (4)		

### Hydrogen-bond geometry (Å, °)

**Table:**

*D*—H···*A*	*D*—H	H···*A*	*D*···*A*	*D*—H···*A*
O4—H4···N	0.82	2.17	2.964 (4)	164
C1—H1···O4^i^	0.98	2.57	3.533 (4)	167
C11—H11···O1^ii^	0.98	2.50	3.403 (4)	154

Symmetry codes:  (i) *x*+1/2, −*y*+5/2, −*z*; (ii) *x*−1/2, −*y*+3/2, −*z*.
